# Factors affecting locoregional recurrence in breast cancer patients undergoing surgery following neoadjuvant treatment

**DOI:** 10.1186/s12893-021-01158-7

**Published:** 2021-03-23

**Authors:** Hsu-Huan Chou, Wei-Shan Chung, Rong-Yao Ding, Wen-Ling Kuo, Chi-Chang Yu, Hsiu-Pei Tsai, Shih-Che Shen, Chia-Hui Chu, Yung-Feng Lo, Shin-Cheh Chen

**Affiliations:** 1 Department of General Surgery, Chang Gung Memorial Hospital, Linkou, No. 5, Fuxing St., Guishan Dist., Taoyuan City, 333 Taiwan; 2 Graduate Institute of Clinical Medical Sciences, College of Medicine, Chang Gung University, No. 259, Wenhua 1st Rd., Guishan Dist., Taoyuan City, 333 Taiwan; 3 Colleges of Medicine, Chang Gung University, No. 259, Wenhua 1st Rd., Guishan Dist., Taoyuan City, 333 Taiwan

**Keywords:** Breast cancer, Neoadjuvant chemotherapy, Locoregional recurrence, Hormone receptor

## Abstract

**Background:**

Neoadjuvant chemotherapy (NAC) has been the standard treatment for locally advanced breast cancer for the purpose of downstaging or for conversion from mastectomy to breast conservation surgery (BCS). Locoregional recurrence (LRR) rate is still high after NAC. The aim of this study was to determine predictive factors for LRR in breast cancer patients in association with the operation types after NAC.

**Methods:**

Between 2005 and 2017, 1047 breast cancer patients underwent BCS or mastectomy after NAC in Chang Gung Memorial Hospital, Linkou. We obtained data regarding patient and tumor characteristics, chemotherapy regimens, clinical tumor response, tumor subtypes and pathological complete response (pCR), type of surgery, and recurrence.

**Results:**

The median follow-up time was 59.2 months (range 3.13–186.75 months). The mean initial tumor size was 4.89 cm (SD ± 2.95 cm). Of the 1047 NAC patients, 232 (22.2%) achieved pCR. The BCS and mastectomy rates were 41.3% and 58.7%, respectively. One hundred four patients developed LRR (9.9%). Comparing between patients who underwent BCS and those who underwent mastectomy revealed no significant difference in the overall LRR rate of the two groups, 8.8% in BCS group vs 10.7% in mastectomy group (*p* = 0.303). Multivariate analysis indicated that independent factors for the prediction of LRR included clinical N2 status, negative estrogen receptor (ER), and failure to achieve pCR. In subgroups of multivariate analysis, only negative ER was the independent factor to predict LRR in mastectomy group (*p* = 0.025) and hormone receptor negative/human epidermal growth factor receptor 2 positive (HR−/HER2 +) subtype (*p* = 0.006) was an independent factor to predict LRR in BCS patients. Further investigation according to the molecular subtype showed that following BCS, non-pCR group had significantly increased LRR compared with the pCR group, in HR−/HER2 + subtype (25.0% vs 8.3%, *p* = 0.037), and HR−/HER2− subtype (20.4% vs 0%, *p* = 0.002).

**Conclusion:**

Clinical N2 status, negative ER, and failure to achieve pCR after NAC were independently related to the risk of developing LRR. Operation type did not impact on the LRR. In addition, the LRR rate was higher in non-pCR hormone receptor-negative patients undergoing BCS comparing with pCR patients.

## Background

Neoadjuvant chemotherapy (NAC) has generally been accepted as the standard treatment for locally advanced breast cancer. With the aim of making non-operable breast cancer operable, NAC increases the number of breast conservative therapy (BCS) candidates by downsizing locally advanced tumor [1, 2]. Hence, patients undergoing BCS after NAC might have a more favorable cosmetic outcome and quality of life [3].

Despite the fact that increased use of NAC has facilitated the treatment of breast cancer, long-term optimized outcomes and treatment effects are concerning. In their recently published meta-analysis on NAC and adjuvant chemotherapy, Asselain and colleagues reported that NAC has a higher local recurrence rate than adjuvant chemotherapy in patients with locally advanced tumors but no significant difference in terms of overall survival between patients undergoing NAC and adjuvant chemotherapy [4]. Similar results were reported in a meta-analysis by Mauri in 2005 [5]. The frequency of BCS, defined as lumpectomy, with whole breast irradiation, is higher after the use of NAC compared with adjuvant chemotherapy after surgery [4].

The long-term survival rate among early breast cancer women without NAC who undergo BCS was the same as that among women who undergo radical mastectomy [6, 7]. However, several studies have demonstrated no significant difference in long-term survival or local recurrence rates between women who underwent BCS and those who underwent radical mastectomy after NAC [8, 9]. Although predictive factors for local recurrence in these NAC-treated patient groups have not yet been clarified yet, the higher local recurrence risk of NAC than of adjuvant chemotherapy is controversial.

NAC is increasingly used in breast cancer therapy, and clinic-pathological subtypes, such as estrogen receptor positive (ER), progesterone receptor positive (PR), and human epidermal growth factor receptor 2 positive (HER-2), can provide prognostic information regarding the risk of local regional recurrence after NAC. Furthermore, pathological complete response (pCR) after NAC can significantly affect the prognosis of breast cancer [10, 11]. We would like to investigate these attributed factors for recurrence reported in retrospective studies on NAC. Furthermore, once the pCR-achieved status is confirmed, the omission of mastectomy is feasible. Patients receiving NAC, in total, have higher BCS rate in literature. We investigated these clinic-pathological attributed factors in association with the operation types after NAC.

## Methods

The study was a retrospective cohort study and was approved by ethics committee of our institution and institutional review board number was 1711150042. Female patients diagnosed with histologically proven unilateral invasive breast cancer who received neo-adjuvant chemotherapy (NAC) and underwent mastectomy or breast conserving surgery at Chang Gung Memorial Hospital, Linkou, between 2005 and 2017 were enrolled. Exclusion criteria were inflammatory cancer, the initial presence of distant metastasis, synchronous bilateral breast cancer, loss of clinical information, and patients who did not undergo surgery after NAT either due to loss of follow up or due to progression development of metastasis after NAT. Clinical staging was determined through physical examination, mammography, ultrasonography of the breast and axillary lymph nodes, a bone scan, and a whole body computed tomography (CT) scan. Histological Diagnosis was confirmed by the specimen of core needle biopsy and abnormal axillary lymph node was routinely evaluated by fine needle aspiration. Clinical information including age, tumor histology, molecular subtype, TNM stage, Scarff-Bloom-Richardson (SBR) grade, NAC regimens, post-NAC response status, operation type, and locoregional recurrence were collected. Chemotherapy regimens included anthracycline-based and taxane-based regimens and the target therapy for HER2 contained trastuzumab and pertuzumab for some patients. Dose modifications were based on blood cell counts and adverse events. Preoperative radiological evaluation included breast ultrasonography and mammography. The range of resection would be decided as original tumor size while the scattered distribution of the tumor was observed after response of the treatment; the range of resection would be the new tumor size while concentric response after NAC according to the guideline of our institution. Operation including mastectomy or BCS and axillary sentinel lymph node biopsies or axillary lymph node dissections according to clinical posttreatment evaluation were performed 2–4 weeks after NAC was completed. The indication for postoperative radiotherapy included all patients after BCS and the patients receiving mastectomy with initial T3 stage (tumor size > 5 cm) and initial N2 stage or residual positive lymph node after NAC. Almost all patients received postoperative adjuvant therapy, including hormone therapy, chemotherapy, and target therapy, according to their clinicopathological factors. Analysis of estrogen receptor, progesterone receptor, HER2 expression and the definition of pCR were described below. The analysis was performed using immunohistochemistry (IHC) staining techniques on pretreatment core needle biopsy specimens. For ER and PR, the positivity in > 1% of tumor cells was defined as expression. Hormone receptor (HR) positive was defined as ER or PR positive while HR negative was categorized as both negative ER and PR. The definition of positive HER2 was IHC staining with a score of 3 + or a positive fluorescence in situ hybridization (FISH) test with IHC staining with a score of 2 + . pCR was defined as no residual invasive cancer cells in the breast and axillary lymph nodes (ypT0/ypTisN0). LRR was defined as ipsilateral breast tumor recurrence including invasive carcinoma and carcinoma in situ, chest wall or skin recurrence, and ipsilateral regional lymph node recurrence including the axilla, internal mammary and supraclavicular region.

### Statistical analysis

Numerical data were compared using a Student’s t-test and presented as the mean + standard deviation (SD) and a two-tailed *p*-value < 0.05 was considered to be statistically significant. Pearson’s chi-squared test for determining whether measurements from different groups are independent (the expected value in each cell is greater than 5), if an expected number is less than 5- > use Fisher's exact test of independence. To compare proportions of a categorical outcome according to different independent groups, we use the chi-squared test or Fisher’s exact test when appropriate. A log-rank test and the Kaplan–Meier method were used for survival analysis. For multivariate analysis of prognostic factors, logistic regression was used. All analyses were performed using SPSS 22.0 for Windows (SPSS, Chicago, IL).

## Results

During the study period, we enrolled 1047 consecutive patients with breast cancer who were treated with NAC followed by surgery and adjuvant therapy. The median follow-up time was 59.2 months (range 3.13–186.75 months) and the last follow up date was June 30, 2020. The mean initial tumor size was 4.89 cm (SD ± 2.95 cm). The T2 stage accounted for 582 patients (55.6%), T3 for 205 patients (19.6%), and T4 for 220 patients (21.0%). For the clinical lymph node status, 79 patients (7.5%) were N0, 543 (51.9%) were N1, 425 (40.6%) were N2. Overall, 1028 (98%) were diagnosed with invasive ductal carcinoma, of whom 77 patients (7.3%) had tumor SBR grade 1, 405 (38.7%) had grade 2 for, and 466 (44.5%) had grade 3. According to IHC staining, 413 (39.4%) patients were ER-negative, while 634 patients (60.6%) were ER-positive; 520 (49.7%) patients were PR-negative, while 527 (50.3%) were PR-positive; and 466 (44.5%) patients were HER-2 positive. The triple negative subtype (HR−/HER2−) accounted for 15.5% (n = 162) of patients, and the HR + /HER2− subtype accounted for 40.0% (n = 419). Regimens for NAC was prescribed according to the physician’s preference. A total of 603 patients (57.7%) were prescribed anthracycline-based agents combined with taxane-based regimens, while 325 patients (31.0%) received chemotherapy agents combined with target therapies, of whom 68 patients received dual blockade therapy for HER2 + disease. Out of a total of 68 HER2 + patients receiving dual blockade therapy (trastuzumab + pertuzumab), 34 patients (50%) achieved pCR while pCR occurred in 104 patients (40.5%) out of 257 HER2 + patients with single blockade therapy (*p* = 0.1579). All patients underwent surgery after NAC; the BCS rate was 41.3% (n = 432), and the mastectomy rate was 58.7% (n = 615). The overall pCR rate was 22.2% (n = 232), while the non-pCR rate was 77.8% (n = 815) (Table 1).

**Table 1:** Clinicopathological characteristics of the study population

Parameters	No. of cases
Age (years), median (IQR)	49 (14)
Tumor size (cm), median (IQR)	4.1 (2.3)
Clinical T stage	
T1	40 (3.8%)
T2	582 (55.6%)
T3	205 (19.6%)
T4	220 (21.0%)
Clinical lymph node status	
N0	79 (7.5%)
N1	543 (51.9%)
N2	425 (40.6%)
Histologic type	
Invasive ductal carcinoma	1028 (98.1%)
Mucinous carcinoma	4 (0.4%)
Invasive lobular carcinoma	10 (1.0%)
Invasive micropapillary carcinoma	5 (0.5%)
SBR grade	
1	77 (7.3%)
2	405 (38.7%)
3	466 (44.5%)
Unknown	99 (9.5%)
ER status	
Negative	413 (39.4%)
Positive	634 (60.6%)
PR status	
Negative	520 (49.7%)
Positive	527 (50.3%)
HER2 status	
Negative	581 (55.5%)
Positive	466 (44.5%)
Subtype	
HR + /HER2−	419 (40.0%)
HR + /HER2 +	237 (22.6%)
HR−/HER2 +	229 (21.9%)
HR−/HER2−	162 (15.5%)
Surgery	
Mastectomy	615 (58.7%)
Breast conserving surgery	432 (41.3%)
Margin	
Free	1014 (96.8%)
Positive	33 (3.2%)
Neoadjuvant regimens	
Anthracycline only	37 (3.5%)
Taxane only	82 (7.8%)
Antracycline + taxane	603 (57.7%)
Chemotherapy + target therapy	325 (31.0%)
Pathological response	
pCR	232 (22.2%)
Non-pCR	815 (77.8%)

*ER* estrogen receptor, *HR* hormone receptor, *HER-2* human epidermal growth factor receptor 2, *pCR* pathological complete response, *PR* progesterone receptor

Clinical T stage (*p* < 0.0001), clinical N stage (*p* < 0.0001), SBR grade (*p* < 0.0001), negative ER/PR (*p* < 0.0001), positive HER2 (*p* < 0.0001) and molecular subtype (*p* < 0.0001) were predictors of pCR in univariate analysis (Table 2). Multivariate analysis found similar results, including clinical T1 stage (*p* < 0.001) and clinical T2 (*p* = 0.028) as predictors of pCR compared with clinical T4 stage. Clinical N0 (*p* = 0.014) and clinical N1 (*p* < 0.0001) were also predictors of pCR compared with clinical N2 stage. SBR grade 3 (*p* = 0.033) was predictor of pCR compared with SBR grade 1. With regard to subtype classification, HR + /HER2 + (*p* < 0.0001), HR−/HER2 + (*p* < 0.0001) and HR−/HER2− (*p* < 0.0001) were predictors of pCR (Table 3).

**Table 2:** Comparison between patients pCR and non-pCR

Parameters	pCR	non-pCR	P value
	(n = 232)	(n = 815)	
Age (years)			0.493
≦50	132 (23.0%)	443 (77.0%)	
> 50	100 (21.2%)	372 (78.8%)	
Clinical T stage			< 0.0001
T1	17 (42.5%)	23 (57.5%)	
T2	149 (25.6%)	433 (74.4%)	
T3	39 (19.0%)	166 (81.0%)	
T4	27 (12.3%)	193 (87.7%)	
Clinical lymph node status			< 0.0001
N0	19 (24.1%)	60 (75.9%)	
N1	164 (30.2%)	379 (69.8%)	
N2	49 (11.5%)	376 (88.5%)	
SBR grade			< 0.0001
1	4 (5.2%)	73 (94.8%)	
2	51 (12.6%)	354 (87.4%)	
3	110 (23.6%)	356 (76.4%)	
Unknown	67 (67.7%)	32 (32.3%)	
ER status			< 0.0001
Negative	144 (32.9%)	269 (65.1%)	
Positive	88 (13.9%)	546 (86.1%)	
PR status			< 0.0001
Negative	172 (33.1%)	348 (66.9%)	
Positive	60 (11.4%)	467 (88.6%)	
HER2 status			< 0.0001
Negative	78 (13.4%)	503 (86.6%)	
Positive	154 (33.0%)	312 (67.0%)	
Subtype			< 0.0001
HR + /HER2−	27 (6.4%)	392 (93.6%)	
HR + /HER2 +	66 (27.8%)	171 (72.2%)	
HR−/HER2 +	88 (38.4%)	141 (61.6%)	
HR−/HER2−	51 (31.5%)	111 (68.5%)	

*ER* estrogen receptor, *HR* hormone receptor, *HER-2* human epidermal growth factor receptor 2, *pCR* pathological complete response

**Table 3:** Multivariate analysis of factors predicting pCR after NAC

Parameters	Odds ratio	95% Confidence interval	P value
Clinical T stage			
T1 vs T4	5.304	2.222–12.664	< 0.001
T2 vs T4	1.808	1.065–3.067	0.028
T3 vs T4	1.216	0.648–2.282	0.541
Clinical N stage			
N0 vs N2	2.434	1.201–4.933	0.014
N1 vs N2	3.344	2.196–5.090	< 0.0001
SBR grade			
2/1	2.327	0.765–7.073	0.137
3/1	3.312	1.101–9.963	0.033
Subtype			
HR + /HER2 + vs HR + /HER2−	4.851	2.862–8.221	< 0.0001
HR−/HER2 + vs HR + / HER2−	8.781	5.146–14.986	< 0.0001
HR−/HER2− vs HR + /HER2−	4.868	2.722–8.706	< 0.0001

*HR* hormone receptor, *HER-2* human epidermal growth factor receptor 2, *NAC* neoadjuvant chemotherapy, *pCR* pathological complete response

**Fig. 1:**
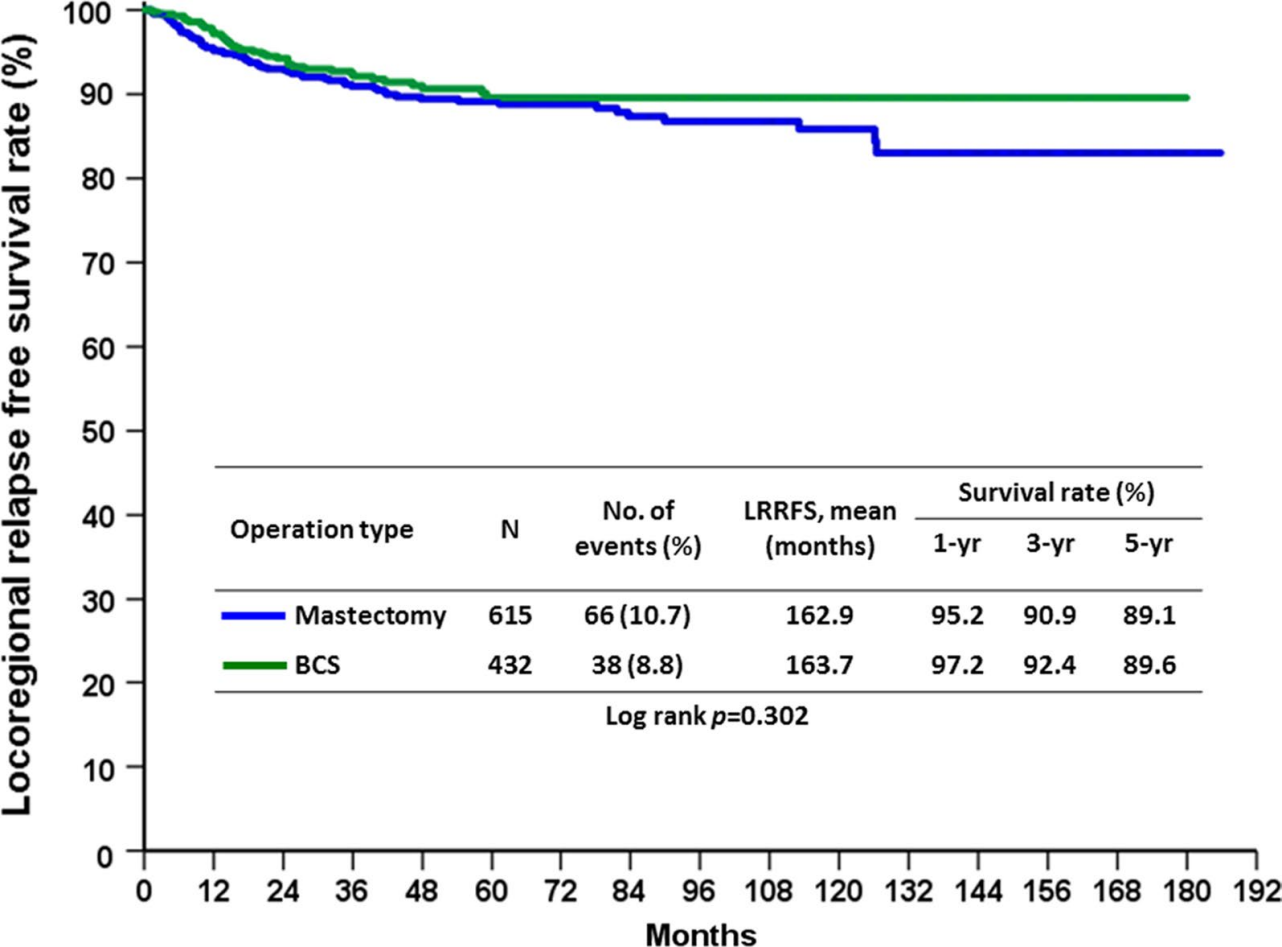
LRRFS rate in the BCS group compared with the mastectomy group

**Fig. 2:**
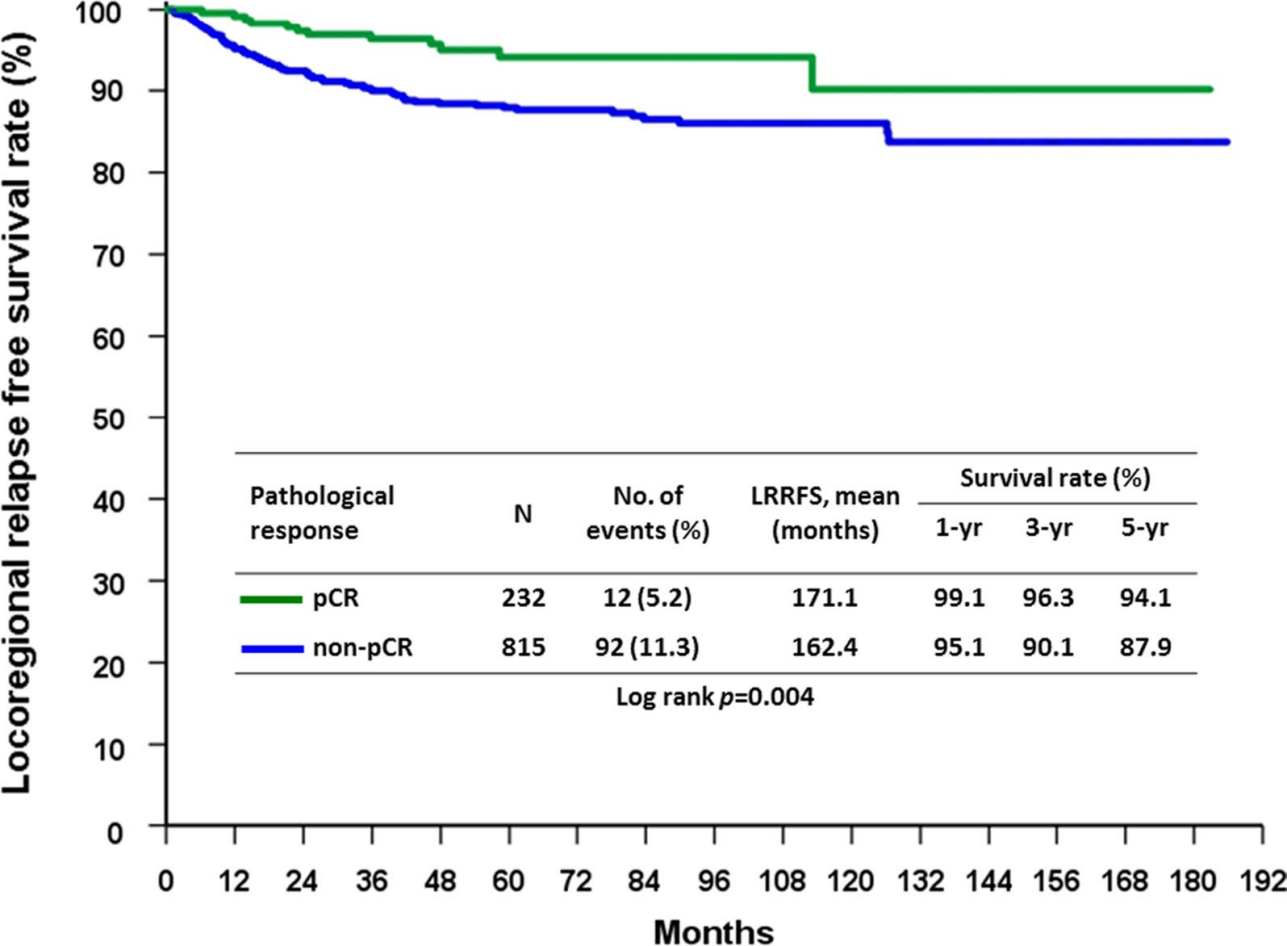
LRRFS rate in the pCR group compared with the non-pCR group

During the follow up period, the recurrence rate was 26.8% (n = 281), of which 9.9% (n = 104) was locoregional recurrence (LRR) and 20.9% was distal metastasis. By the conclusion of this study, 191 (18.2%) patients had died from breast cancer and 23 (2.2%) patients had died from other causes. Furthermore, on univariate analysis of factors affecting LRR, we noted that clinical N2 stage (*p* < 0.0001), SBR grade 3 (*p* = 0.004), negative ER (*p* = 0.001), negative PR (*p* = 0.003), negative HR subtypes (*p* = 0.043), and non-pCR (*p* = 0.006) were predictors of LRR (Table 4). Comparing between patients who underwent BCS and those who underwent mastectomy revealed no significant difference in the overall LRR rate of the two groups, 8.8% in BCS group vs 10.7% in mastectomy group (*p* = 0.303). In multivariate analysis, only clinical N2 status (*p* = 0.018), negative ER (*p* = 0.011) and non-pCR (*p* = 0.019) were found to be predictors of LRR (Table 5). No significant difference of locoregional recurrence free survival rate (LRRFS) in the BCS and mastectomy groups are shown in Fig. 1 (*p* = 0.302). The effect of pCR on LRRFS is demonstrated in Fig. 2 (*p* = 0.004), as 5-year LRRFS is 94.1% in pCR group and 87.9% in non-pCR group.

**Table 4:** Factors predicting LRR after NAC

Parameters	LRR	No LRR	P value
	(n = 104)	(n = 943)	
Age (years)			0.66
≦50	55 (9.6%)	520 (90.4%)	
> 50	49 (10.4%)	423 (89.6%)	
Clinical T stage			0.059
T1	5 (12.5%)	35 (87.5%)	
T2	51 (8.8%)	531 (91.2%)	
T3	16 (7.8%)	189 (92.2%)	
T4	32 (14.5%)	188 (85.5%)	
Clinical lymph node status			< 0.0001
N0	2 (2.5%)	77 (97.5%)	
N1	40 (7.4%)	503 (92.6%)	
N2	62 (14.6%)	363 (85.4%)	
SBR grade			0.004
1	2 (2.6%)	75 (97.4%)	
2	38 (9.4%)	367 (90.6%)	
3	60 (12.9%)	406 (87.1%)	
Unknown	4 (4.0%)	95 (96.0%)	
Histologic type			0.083
Invasive ductal carcinoma	100 (9.7%)	928 (90.3%)	
Invasive lobular carcinoma	2 (20.0%)	8 (80.0%)	
Mucinous carcinoma	0	4 (100.0%)	
Others	2 (40.0%)	3 (60.0%)	
Margin			0.765
Free	102 (10.1%)	912 (89.9%)	
Positive	2 (6.1%)	31 (93.9%)	
ER			0.001
Positive	47 (7.4%)	587 (92.6%)	
Negative	57 (13.8%)	356 (86.2%)	
PR			0.003
Positive	38 (7.2%)	489 (92.8%)	
Negative	66 (12.7%)	454 (87.3%)	
HER2			0.722
Positive	48 (10.3%)	418 (89.7%)	
Negative	56 (9.6%)	525 (90.4%)	
Subtype			0.043
HR + /HER2−	33 (7.9%)	386 (92.1%)	
HR + /HER2 +	19 (8.0%)	218 (92.0%)	
HR−/HER2 +	29 (12.7%)	200 (87.3%)	
HR−/HER2−	23 (14.2%)	139 (85.8%)	
Neoadjuvant regimens			0.189
Anthracycline only	7 (18.9%)	30 (81.1%)	
Taxane only	6 (7.3%)	76 (92.7%)	
Anthracycline + taxane	63 (10.4%)	540 (89.6%)	
Chemotherapy + target therapy	28 (8.6%)	297 (91.4%)	
Operation type			0.303
Mastectomy	66 (10.7%)	549 (89.3%)	
BCS	38 (8.8%)	394 (91.2%)	
pCR			0.006
Yes	12 (5.2%)	220 (94.8%)	
No	92 (11.3%)	723 (88.7%)	

*ER* estrogen receptor, *HR* hormone receptor, *HER-2* human epidermal growth factor receptor 2, *LRR* locoregional recurrence, *NAC* neoadjuvant chemotherapy, *pCR* pathological complete response, *PR* progesterone receptor

**Table 5:** Multivariate analysis of factors to predict LRR

Parameters	Odds ratio	95% Confidence interval	P value
Clinical lymph node status			
N0	1		
N1	2.981	0.700–12.701	0.14
N2	5.668	1.343–23.929	0.018
SBR grade			
1	1		
2	3.012	0.700–12.954	0.139
3	3.572	0.827–15.428	0.088
Unknown	1.541	0.259–9.157	0.634
ER			
Positive	1		
Negative	4.272	1.405–12.995	0.011
PR			
Positive	1		
Negative	1.768	0.896–3.487	0.1
Subtype			
HR + /HER2−	1		
HR + /HER2 +	1.029	0.552–1.919	0.929
HR−/HER2 +	0.295	0.075–1.159	0.08
HR−/HER2−	0.329	0.083–1.313	0.115
pCR			
Yes	1		
No	2.247	1.146–4.407	0.019

*HR* hormone receptor, *HER-2* human epidermal growth factor receptor 2, *LRR* locoregional recurrence, *pCR* pathological complete response

**Fig. 3:**
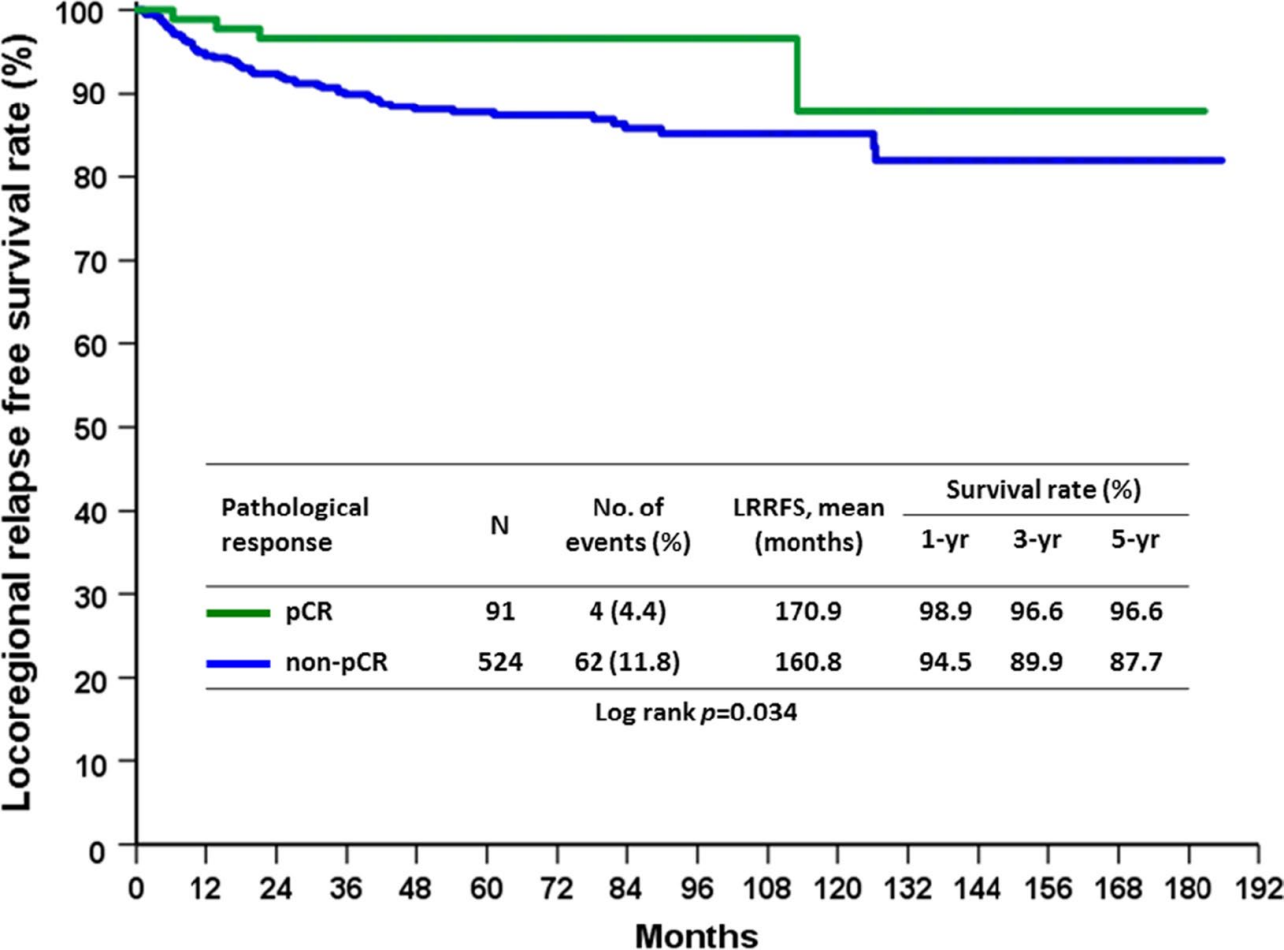
LRRFS rate in the pCR group compared with the non-pCR group in patients who underwent mastectomy

**Fig. 4:**
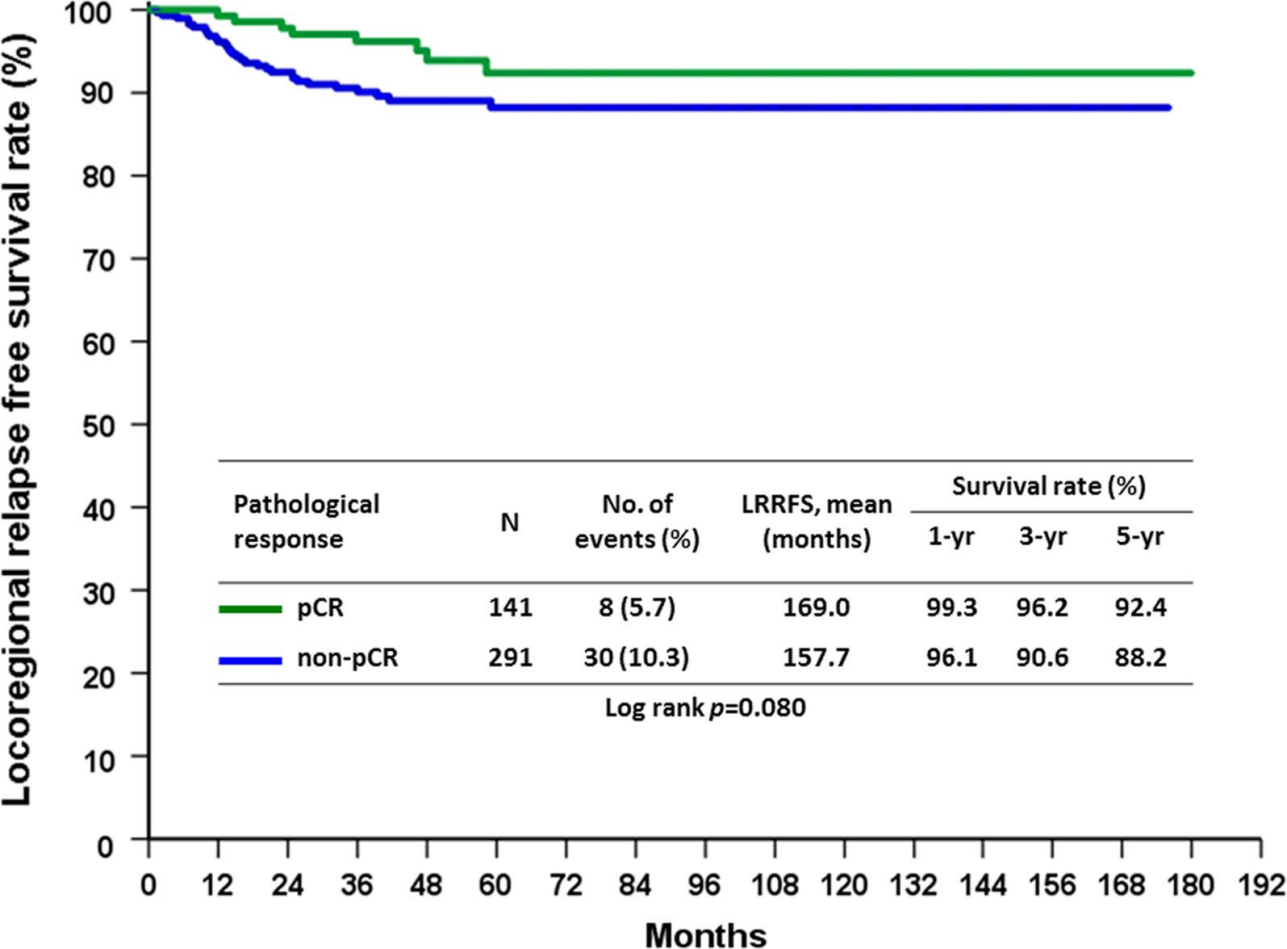
LRRFS rate in the pCR group compared with the non-pCR group in patients who underwent BCS

In patients who underwent mastectomy, clinical T stage (*p* = 0.019), clinical N stage (*p* < 0.001), negative ER (*p* = 0.032) and non-pCR (*p* = 0.034) were factors to predict LRR in univariate analysis. In multivariate analysis, only negative ER remained an independent factor for unfavorable LRR compared with ER positive patients in mastectomy group (*p* = 0.025) (Table 6). The LRRFS rate in the pCR group following mastectomy showed a favorable outcome (*p* = 0.034), as shown in Fig. 3, while 5-year LRRFS was 92.4% in pCR group and 88.2% in non-pCR group. In BCS group, negative ER (*p* = 0.005), negative PR (0.004) and negative HR molecular subtype (*p* = 0.029) were predictors to LRR in univariate analysis. HR−/HER2 + subtype (*p* = 0.006) was an independent factor to predict LRR in BCS patients (Table 7). LRRFS in BCS group was also found to be numerically, but not significant, different between the pCR and non-pCR groups, as shown in Fig. 4 (*p* = 0.080). Further analysis focusing on the subtypes of the BCS group demonstrated that the HR−/HER2 + non-pCR group had significantly increased LRR compared with the HR−/HER2 + pCR group (25.0% vs 8.3%, *p* = 0.037). Furthermore, the HR−/HER2−non-pCR group had significantly increased LRR compared with the HR−/HER2−pCR group (20.4% vs 0%, *p* = 0.002). (Table 8). Moreover, there was no difference in LRR found with regards to hormone receptor positive disease between the between pCR and non-pCR groups. Further analysis of molecular subtypes indicated no significant difference for all subtypes between the pCR and non-pCR groups following mastectomy (Table 8).

**Table 6:** Univariate and multivariate analysis of factors predicting LRR in patients undergoing mastectomy after NAC

Parameters	Univariate analysis			Multivariate analysis		
	LRR (n = 66)	No LRR (n = 549)	P value	Odds ratio	95% Confidence interval	P value
Age (years)			0.477			
≦50	30 (9.8%)	275 (90.2%)				
> 50	36 (11.6%)	274 (88.4%)				
Clinical T stage			0.019			
T1	3 (20.0%)	12 (80.0%)		1		
T2	23 (9.1%)	229 (90.9%)		0.335	0.081–1.380	0.13
T3	9 (6.1%)	138 (93.9%)		0.228	0.050–1.035	0.055
T4	31 (15.4%)	170 (84.6%)		0.434	0.105–1.782	0.246
Clinical lymph node status			< 0.001			
N0	1 (4.8%)	20 (95.2%)		1		
N1	16 (5.7%)	264 (94.3%)		1.223	0.151–9.914	0.85
N2	49 (15.6%)	265 (84.4%)		2.983	0.380–23.421	0.299
SBR grade			0.07			
1	1 (2.1%)	46 (97.9%)				
2	25 (10.3%)	217 (89.7%)				
3	37 (13.5%)	238 (86.5%)				
Unknown	3 (5.9%)	48 (94.1%)				
Histologic type			0.534			
Invasive ductal carcinoma	64 (10.6%)	539 (89.4%)				
Invasive lobular carcinoma	1 (16.7%)	5 (83.3%)				
Mucinous carcinoma	0	3 (100.0%)				
Others	1 (33.3%)	2 (66.7%)				
Margin			> 0.999			
Free	66 (10.1%)	541 (89.1%)				
Positive	0	8 (100.0%)				
ER			0.032			
Positive	33 (8.6%)	349 (91.4%)		1		
Negative	33 (14.2%)	200 (85.8%)		1.837	1.078–3.131	0.025
PR			0.105			
Positive	29 (8.8%)	299 (91.2%)				
Negative	37 (12.9%)	250 (87.1%)				
HER2			0.51			
Positive	27 (9.8%)	248 (90.2%)				
Negative	39 (11.5%)	301 (88.5%)				
Subtype			0.206			
HR + /HER2−	26 (9.8%)	240 (90.2%)				
HR + /HER2 +	11 (8.5%)	119 (91.5%)				
HR−/HER2 +	16 (11.0%)	129 (89.0%)				
HR−/HER2−	13 (17.6%)	61 (82.4%)				
Neoadjuvant regimens			0.229			
Anthracycline only	5 (19.2%)	21 (80.8%)				
Taxane only	5 (11.4%)	39 (88.6%)				
Anthracycline + Taxane	44 (11.6%)	336 (88.4%)				
Chemotherapy + Target therapy	12 (7.3%)	153 (92.7%)				
pCR			0.034			
Yes	4 (4.4%)	87 (95.6%)		1		
No	62 (11.8%)	462 (88.2%)		2.524	0.856–7.444	0.093

*ER* estrogen receptor, *HR* hormone receptor, *HER-2* human epidermal growth factor receptor 2, *LRR* locoregional recurrence, *NAC*, neoadjuvant chemotherapy, *pCR* pathological complete response, *PR* progesterone receptor

**Table 7:** Univariate and multivariate analysis of factors predicting LRR in patients undergoing BCS after NAC

Parameters	Univariate analysis			Multivariate analysis		
	LRR (n = 38)	No LRR (n = 394)	P value	Odds ratio	95% confidence interval	P value
Age (years)			0.661			
≦50	25 (9.3%)	245 (90.7%)				
> 50	13 (8.0%)	149 (92.0%)				
Clinical T stage			0.77			
T1	2 (8.0%)	23 (92.0%)				
T2	28 (8.5%)	302 (91.5%)				
T3	7 (12.1%)	51 (87.9%)				
T4	1 (5.3%)	18 (94.7%)				
Clinical lymph node status			0.089			
N0	1 (1.7%)	57 (98.3%)				
N1	24 (9.1%)	239 (90.9%)				
N2	13 (11.7%)	98 (88.3%)				
SBR grade			0.091			
1	1 (3.3%)	29 (96.7%)				
2	13 (8.0%)	150 (92.0%)				
3	23 (12.0%)	168 (88.0%)				
Unknown	1 (2.1%)	47 (97.9%)				
Histologic type			0.127			
Invasive ductal carcinoma	36 (8.5%)	389 (91.5%)				
Invasive lobular carcinoma	1 (25.0%)	3 (75.0%)				
Mucinous carcinoma	0	1 (100.0%)				
Others	1 (50.0%)	1 (50.0%)				
Margin			> 0.999			
Free	36 (8.8)	371 (91.2%)				
Positive	2 (8.0%)	23 (92.0%)				
ER			0.005			
Positive	14 (5.6%)	238 (94.4%)				
Negative	24 (13.3%)	156 (86.7%)				
PR			0.004			
Positive	9 (4.5%)	190 (95.5%)				
Negative	29 (12.4%)	204 (87.6%)				
HER2			0.151			
Positive	21 (11.0%)	170 (89.0%)				
Negative	17 (7.1%)	224 (92.9%)				
Subtype			0.029			
HR + /HER2−	7 (4.6%)	146 (95.4%)		1		
HR + /HER2 +	8 (7.5%)	99 (92.5%)		1.685	0.592–4.797	0.328
HR-/HER2 +	13 (15.5%)	71 (84.5%)		3.819	1.460–9.990	0.006
HR−/HER2−	10 (11.4%)	78 (88.6%)		2.674	0.980–7.300	0.055
Neoadjuvant regimens			0.345			
Anthracycline only	2 (18.2%)	9 (81.8%)				
Taxane only	1 (2.6%)	37 (97.4%)				
Anthracycline + taxane	19 (8.5%)	204 (91.5%)				
Chemotherapy + target therapy	16 (10.0%)	144 (90.0%)				
pCR			0.111			
Yes	8 (5.7%)	133 (94.3%)				
No	30 (10.3%)	261 (89.7%)				

*ER* estrogen receptor; *HR* hormone receptor; *HER-2* human epidermal growth factor receptor 2; *LRR* locoregional recurrence; *NAC* neoadjuvant chemotherapy; *pCR* pathological complete response; *PR* progesterone receptor

**Table 8:** Comparison of LRR in pCR and non-pCR patients after BCS and mastectomy in different subtypes

Parameters	Subtype	Recurrence patterns	Pathological total response		
Operation type			pCR	Non-pCR	P value
			(N = 91)	(N = 524)	
Mastectomy	HR + /HER2-	LRR	1 (9.1%)	25 (9.8%)	> 0.999
(N = 615)	(N = 266)	No LRR	10 (90.9%)	230 (90.2%)	
	HR + /HER2 +	LRR	0	11 (10.8%)	0.12
	(N = 130)	No LRR	28 (100%)	91 (89.2%)	
	HR−/HER2 +	LRR	1 (2.5%)	15 (14.3%)	0.071
	(N = 145)	No LRR	39 (97.5%)	90 (85.7%)	
	HR−/HER2−	LRR	2 (16.7%)	11 (17.7%)	> 0.999
	(N = 74)	No LRR	10 (83.3%)	51 (82.3%)	
Conserving breast surgery	HR + /HER2−	LRR	1 (6.3%)	6 (4.4%)	0.546
(n = 432)	(N = 153)	No LRR	15 (93.7%)	131 (95.6%)	
	HR + /HER2 +	LRR	3 (7.9%)	5 (7.2%)	> 0.999
	(N = 107)	No LRR	35 (92.1%)	64 (92.8%)	
	HR−/HER2 +	LRR	4 (8.3%)	9 (25.0%)	0.037
	(N = 84)	No LRR	44 (91.7%)	27 (75.0%)	
	HR−/HER2−	LRR	0	10 (20.4%)	0.002
	(N = 88)	No LRR	39 (100%)	39 (79.6%)	

*HR* hormone receptor, *HER-2* human epidermal growth factor receptor 2, *LRR* locoregional recurrence, *pCR* pathological complete response

## Discussion

In total, 232 patients (22.2%) achieved pCR. Multivariate analysis indicated that clinical T stage, clinical N stage, and molecular subtype were independent predictors to pCR. A total of 104 patients (9.9%) developed LRR of which 12 were in the pCR group and 92 were in the non-pCR group. Our study reported that pCR in all breast cancer subtypes, after NAC, provided better local control. The result was in line with the findings of our previous report published in 2018, in which no LRR occurred in the pCR group and 31 patients (13.2%) in the non-pCR group with significant difference in total 263 patients all receiving neoadjuvant weekly epirubicin and docetaxel regimens[12]. Our study revealed that 232 patients (22.2%) achieved pCR among the 1047 patients underwent while the BCS rate is 41% and the rest of patients received mastectomy (59%). Overall, 281 patients experienced tumor recurrence (26.8%).

Although the pooled analysis from CTNeoBC did not support pCR as a surrogate endpoint for an improved event-free survival or overall survival in all subtypes of breast cancer [13], pCR was an effective surrogate endpoint for selected patients in aggressive subtypes including luminal B/HER-, HER2 overexpression and triple negative breast cancer [14]. A recent comprehensive meta-analysis concluded that pCR followed by NAC was associated with significantly better event-free survival (EFS) and overall survival (OS), especially for patients with triple-negative and HER2 + breast cancer. The tumor response effect observed in the pCR group was similar in adjuvant chemotherapy and NAC patients [15]. Moreover, data from the combined analysis of the National Surgical Adjuvant Breast and Bowel Project (NSABP) B-18 and B-27 showed that the residual tumor status was an independent predictor of LRR in all patients at the 10 year follow-up, regardless of surgery type [10]. Another large analysis of the European Organisation for Research and Treatment of Cancer (EORTC) 10,994/BIG 1–00 study of patients with locally advanced breast cancer receiving NAC showed that pCR was a favorable factor with regards to the prediction of LRR after NAC [16]. Several retrospective series also demonstrated that achieving pCR after NAC can result in better local control following surgery [1, 2, 17, 18]. Therefore, achieving pCR was an important factor not only for distant disease control but also for local control. In our series, failure to achieve pCR was also proved to be an independent predictor of LRR in total population. In subgroup analysis of mastectomy patients, the effect of pCR was only demonstrated in univariate analysis while numerically but not significantly lower LRR in pCR group comparing non-pCR group in patients undergoing BCS.

The results from the EORTC 10994/BIG 1–00 study of patients with locally advanced breast cancer receiving NAC showed that breast cancer subtypes, including HER2 + with or without trastuzumab and triple-negative, are predictive factors for high LRR after NAC [16]. Yang et al. reported that 233 stage II-III breast cancer patients treated with NAC, mastectomy, and post-mastectomy radiotherapy had an 8% LRR rate over 5 years with a 62-month median follow-up. The authors concluded that patients with triple-negative breast cancer had the highest LRR rate and those with HR + and HER2 + breast cancer had favorable LRR rates, regardless of NAC response [18]. In other several retrospective studies, molecular subtypes including HER2 + and triple-negative also showed poor LRR in BCS patients [1, 2]. In our study, negative ER was found an independent significant factor for the prediction of LRR, regardless of treatment response. Negative ER remained an independent factor for unfavorable LRR compared with ER positive patients in mastectomy group and HR−/HER2 + subtype was an independent factor to predict LRR in BCS patients. The status of the hormone receptor appears to play a more important role in LRR than the NAC treatment response in our results.

The Early Breast Cancer Trialists’ Collaborative Group (EBCTCG) recently reported that NAC was associated with more frequent local recurrence than that of adjuvant chemotherapy. The 15-year local recurrence rate was reported to be 21.4% for NACT compared with 15.9% for adjuvant chemotherapy from a meta-analysis of individual patient data from 10 randomized trials with average 9 years of follow-up. The study group also found that patients who underwent NAC had an increased frequency of breast-conserving therapy (65%) versus those treated with adjuvant chemotherapy (49%). The largest difference of LRR appeared in planned mastectomy patients and surgery less commonly used patients. The authors concluded tumors downsized by NAC might have higher local recurrence after BCS than tumors of the same dimensions in women who did not undergo NAC. Furthermore, the majority of patients only received anthracycline-based chemotherapy and final enrollment of patients occurred in 2002 [4]. Previous NSABP B-27 trial reports indicated that anthracycline-based regimens with the addition of taxane were associated with higher pCR rates and better local control [19]. Moreover, neoadjuvant chemotherapy plus trastuzumab was shown to be a predictor factor for favorable long-term survival but trastuzumab cannot be used before 2002 [20]. In our studies, the majority of patients received anthracycline-based regimens combined with taxane-based chemotherapy and every patient underwent appropriate surgery. Results from the combined study of NSABP B-18 and B-27 revealed that the 10-year LRR was 12.3% for patients with mastectomy and 10.3% for patients with lumpectomy plus whole breast radiotherapy, indicating no significant difference between the mastectomy and BCS groups following NAC [10]. In the I-SPY trial there was no substantial difference in LRR between the mastectomy and BCS groups, given the fact that the mastectomy group on average had higher clinical staging [21]. A higher breast conservation rate would not increase the LRR rate, which the NSABP B-18 and the EORTC studies have confirmed [22, 23]. In another pooled analysis of 5500 participants, the mastectomy rate in NAC group was found to be lower than in the adjuvant chemotherapy group, without interfering the local control [24]. In our report, 615 (58.7%) patients chose mastectomy, while 432 (41.3%) received BCS. However, the choice of surgery types did not affect the LRR rate—10.7% in mastectomy patients and 8.8% in BCS patients in the total population.

Subgroup analysis revealed 38 cases of LRR (8.8%) following BCS, in which 5.7% in pCR group and 10.3% in non-pCR group. Further investigation according to the molecular subtype showed that in the BCS group, HR−/HER2 + non-pCR patients had significantly increased LRR than HR−/HER2 + pCR patients and that HR−/HER2− non-pCR patients had a significantly increased LRR than HR−/HER2−pCR patients. In the mastectomy group, an increasing trend with regards to the risk of LRR in the non-pCR group was observed, but this was not significant. Caudle et al. reported that HR−/HER2 + and HR-/HER2- patients with a poor response to NAC had worse LRRFS after BCS. Furthermore, the authors found that patients with HR + /HER2− and HR + /HER2 + subtypes had excellent LRRFS, regardless of tumor response to NAC[1]. Another study group from Korea revealed HR−/HER2− subtypes and HER2 + without trastuzumab subtypes predicted higher rates of LRR after NAC and BCT, while A pCR was predictive of improved LRR in HR−/HER2− subtype [2]. Moreover, the I-SPY 1 Trial reported that the 5-year local recurrence risk was 0% for mastectomy and 9% for breast conservation in patients with an excellent response to NAC while the local recurrence rate was 12% for mastectomy and 7% for breast conservation in significant residual disease [21]. Another critical point was the resection area of operation after NAC. In patients who undergo NAC in order to achieve breast-tumor downstaging to enable BCS, the tumor site should be marked with a clip before initiating NAC, and resection of the entire volume of breast tissue originally occupied by tumor is not necessary [25]. However, the difference of resection area influencing local recurrence was still unknown in neoadjuvant setting. Local control appeared to be worse in HR- subtype non-pCR BCS patients after NAC in our study, but the effect on overall survival remains unknown. Further investigation is needed to determine overall survival outcomes.

### Limitation

One of the limitations of this study is that it was a retrospective study from a single institution, which may result in selection bias. This study is also limited by the relatively short follow-up period. Furthermore, not all patients with the same subtype diagnosis received the same chemotherapy regimens.

## Conclusion

Clinical N2 status, negative ER, and failure to achieve pCR after NAC were independently related to the risk of developing LRR. Operation type did not impact on the LRR. In addition, the LRR rate was higher in non-pCR hormone receptor-negative patients undergoing BCS comparing with pCR patients. Some strategies to guide adjuvant treatment and strict follow up are needed in non-pCR hormone receptor-negative patients undergoing BCS after NAC.

## Data Availability

The datasets used and/or analysed during the current study are available from the corresponding author on reasonable request.

## Abbreviations

NAC: Neoadjuvant chemotherapy
BCS: Breast conservation surgery
LRR: Locoregional recurrence
LRRFS: Locoregional recurrence free survival
CR: Pathological complete response
HR: Hormone receptor
HER2: Human epidermal growth factor receptor
